# Localizing EEG Recordings Associated With a Balance Threat During Unexpected Postural Translations in Young and Elderly Adults

**DOI:** 10.1109/TNSRE.2023.3331211

**Published:** 2023-11-16

**Authors:** Zhuo Wang, Valentina Graci, Thomas Seacrist, Allon Guez, Emily A. Keshner

**Affiliations:** Center for Injury Research and Prevention, Children’s Hospital of Philadelphia, Philadelphia, PA 19104 USA.; Department of Electrical and Computer Engineering, Drexel University, Philadelphia, PA 19104 USA; Center for Injury Research and Prevention, Children’s Hospital of Philadelphia, Philadelphia, PA 19104 USA; School of Biomedical Engineering, Science, and Health System, Drexel University, Philadelphia, PA 19104 USA.; Center for Injury Research and Prevention, Children’s Hospital of Philadelphia, Philadelphia, PA 19104 USA.; Department of Electrical and Computer Engineering, Drexel University, Philadelphia, PA 19104 USA; GraceFall Inc., Penn Valley, PA 19702 USA.; Department of Health and Rehabilitation Sciences, Temple University, Philadelphia, PA 19122 USA; GraceFall Inc., Penn Valley, PA 19702 USA.

**Keywords:** Electroencephalography (EEG), balance, startle responses, elderly, prediction, sex differences

## Abstract

Balance perturbations are accompanied by global cortical activation that increases in magnitude when postural perturbations are unexpected, potentially due to the addition of a startle response. A specific site for best recording the response to unexpected destabilization has not been identified. We hypothesize that a single sensor located near to subcortical brainstem mechanisms could serve as a marker for the response to unpredictable postural events. Twenty healthy young (20.8 ± 2.9 yrs) and 20 healthy elder (71.7 ± 4.2 yrs) adults stood upright on a dynamic platform with eyes open. Platform translations (20 cm at 100 cm/s) were delivered in the posterior (29 trials) and anterior (5 catch trials) directions. Active EEG electrodes were located at Fz and Cz and bilaterally on the mastoids. Following platform acceleration onset, 300 ms of EEG activity from each trial was detrended, baseline-corrected, and normalized to the first trial. Average Root-Mean-Square (RMS) values across “unpredictable” and “predictable” events were computed for each channel. EEG RMS responses were significantly greater with unpredictable than predictable disturbances: Cz (p<0.001), Fz (p<0.003), and mastoid (p<0.0001). EEG RMS responses were also significantly greater in elderly than young adults at Cz (p<0.02) and mastoid (p<0.04). A significant effect of sex in the responses at the mastoid sensors (p<0.04) revealed that elderly male adults were principally responsible for the age effect. These results confirm that the cortical activity resulting from an unexpected postural disturbance could be portrayed by a single sensor located over the mastoid bone in both young and elderly adults.

## I. Introduction

FALLS are a leading cause of injury and accidental death with associated medical costs exceeding $55 billion in 2020 [1]. Despite substantial research into fall prevention, 28-49% of older adults in the United States fall every year [2], [3] and fall-related deaths continue to rise [2]. Mortality in the elderly population was found to be significantly higher than in the non-elderly (4.4% vs. 1.6%) after a ground-level fall [4].

Currently, recommended approaches to treatment for instability include exercise interventions and removal of environmental factors that contribute to a trip or slip, such as rugs, walking in the dark, and obstacles [5]. Progressive resistance training has been shown to be beneficial for individuals at risk for fractures as it improves their physical health and quality of life and reduces pain [6]. However, the negative impact of falls and fall-related injuries continues. Moreover, due to fear of falling, many older adults choose to restrict their activity or have their activity restricted by the individuals responsible for their care, which ultimately increases sedentary behavior and reduces quality of life. Because balance and gait impairments can often lead to falls [7], [8], any successful intervention should target these motor behaviors to develop effective fall prevention interventions. This study aims to investigate cortical involvement in reactive balance control, with the goal of developing applications that promote the detection of balance losses to prevent injuries from a fall.

There are devices emerging in the market that aim to mitigate injuries in the event of a fall. These devices measure body motion (e.g., acceleration sensors) to detect falls and trigger the inflation of a protective garment to protect the users from injury. Current fall detection systems feature inertial and ambient sensors. However, they do not accurately distinguish real falls from fall-like activities of daily living (ADLs), such as, bending down, sitting to standing, or getting out of bed [9]. Accelerometers allow for the identification of key time points in the motion of the fall from onset to completion [10], [11], but it is difficult to distinguish between an actual loss of stability and non-fall activities from these signals, especially when using a single sensor [12]. Thus, accelerometer-based sensing can result in false positive (i.e., a non-fall activity recognized as a fall) and false negative (i.e., an actual fall recognized as a non-fall activity) events. Consequently, there is a need for more advanced and precise methods to detect and prevent falls, especially among individuals at higher risk.

Previous research in balance control has extensively examined the reactive responses to postural disturbances and their implications for fall prevention. Balance perturbations have been shown to elicit global cortical activation within 90 ms after perturbation onset [13]. Termed perturbation evoked potentials (PEP), these responses are widely distributed over the frontal, central, and parietal cortex and can be identified through a single electrode site [14]. Unpredictable postural perturbations leading to a loss of balance evoke a cortical response that is not present in the case of predictable perturbations [15]. An unanticipated disturbance of balance produces gamma and theta band EEG related to cortical modulation and sensorimotor integration [16]; studies have demonstrated that the resulting EEG activity exhibits higher power than that observed during anticipated disturbances [17]. This global activation could reflect a summation of events including, but not limited to, anterior cingulate cortex signals thought to relay information regarding error processing [18]; vestibular signals to collect information about linear and angular velocity and acceleration [19], [20]; pre-motor cortex to capture motor plans being implemented to counter a fall [21]; cerebellum and midbrain nuclei to monitor signals of error detection and motor error subtraction [22].

Evidence demonstrates that predictability of the disturbance modifies this EEG activity related to balance adjustments [14], [23]. Since falls are unexpected events that frequently occur following unpracticed perturbations of standing balance, the first response to external balance perturbations likely includes the startle reflex that is superimposed on the postural reaction aimed to avoid falling and injuries [24]. As with postural motor behaviors recorded during unanticipated vs. anticipated disturbances, the addition of the startle reflex is likely to be detected as an increase in response magnitude [25].

Falls are more prevalent and injurious in the elder population, but changes in the PEP responses have not been revealed as a neural substrate for these events. Although cortical PEPs have been reported as slowing in older adults [26], we have found that, even in older adults, subcortical (mastoid sensor) activity emerges with the shorter latencies and decreased variability indicative of a reflex response [24]. In this study we explored whether temporal characteristics of subcortical PEP responses in the elderly were comparable to those of young adults.

We included both predictable and unpredictable balance disturbances in young and elderly men and women to describe how PEP responses might change with aging. The inclusion of young and elderly male and female adults allowed us to examine age and sex-related variations that might contribute to differences in the neural activity related to postural control.

In addition, the optimal measurement site for responses evoked by unanticipated postural events has not yet been identified. Because we are interested in eventually designing a wearable sensor to record early cortical responses to unpredictable events, we explored whether a sensor over the mastoid bone would be a reliable recording site for young and older adults. We hypothesized that the mastoid sensor location would serve as a reliable marker for the PEP response during postural events across the age range.

Finally, we explored whether the identification of the PEP was dependent on a summation of sensor signals or whether it could be identified through a single EEG channel. Prior work with seated postural disturbances has verified that a single EEG sensory site can be used to reliably indicate the onset of a PEP [14]. We hypothesized that PEPs could be reliably identified from a single EEG sensor placed over the mastoid bone.

Our study incorporates different age and sex groups, as well as different sensor locations (cortical and mastoid), with the aim of identifying more reliable physiological indications of a fall for future applications. Specifically, we are concerned with the possibility of integrating a single channel-ear-EEG with existing fall detection devices that utilize motion or ambient sensors. By integrating the single channel-ear-EEG with existing fall detection technologies, we ultimately anticipate improved detection capabilities and a more personalized, discreet approach to fall prevention, promoting the safety and well-being of individuals in various contexts.

## II. Methods

The study protocol was reviewed and approved by the Institutional Review Board of the Children’s Hospital of Philadelphia, and participants were compensated for their participation.

### A. Participants

Participants for this study were recruited through Research-Match.org, a database designed to match individuals with research studies based on their health conditions. A total of forty participants, with twenty individuals classified as young (age 24.6 ± 5.9 yrs, height 172.2 ± 6.9 cm, weight 69.6 ± 10.6 kg) and twenty individuals classified as elder (age 66.9 ± 5.8 yrs, height 169.4 ± 8.4 cm, weight 68.8 ± 10.3 kg). Each group consisted of ten males and ten females. Exclusion criteria for participation were weight over 195 lbs, cognitive impairment, use of walkers or walking aids, history of the spine, pelvis, or lower extremity fracture in the last 5 years, pregnancy, uncorrected vision or hearing, significant foot deformities or amputation, hip or knee replacement, peripheral neuropathy, use of medication affecting postural control, and fear of falling or amusement parks.

### B. Procedures

**Fig. 1.**
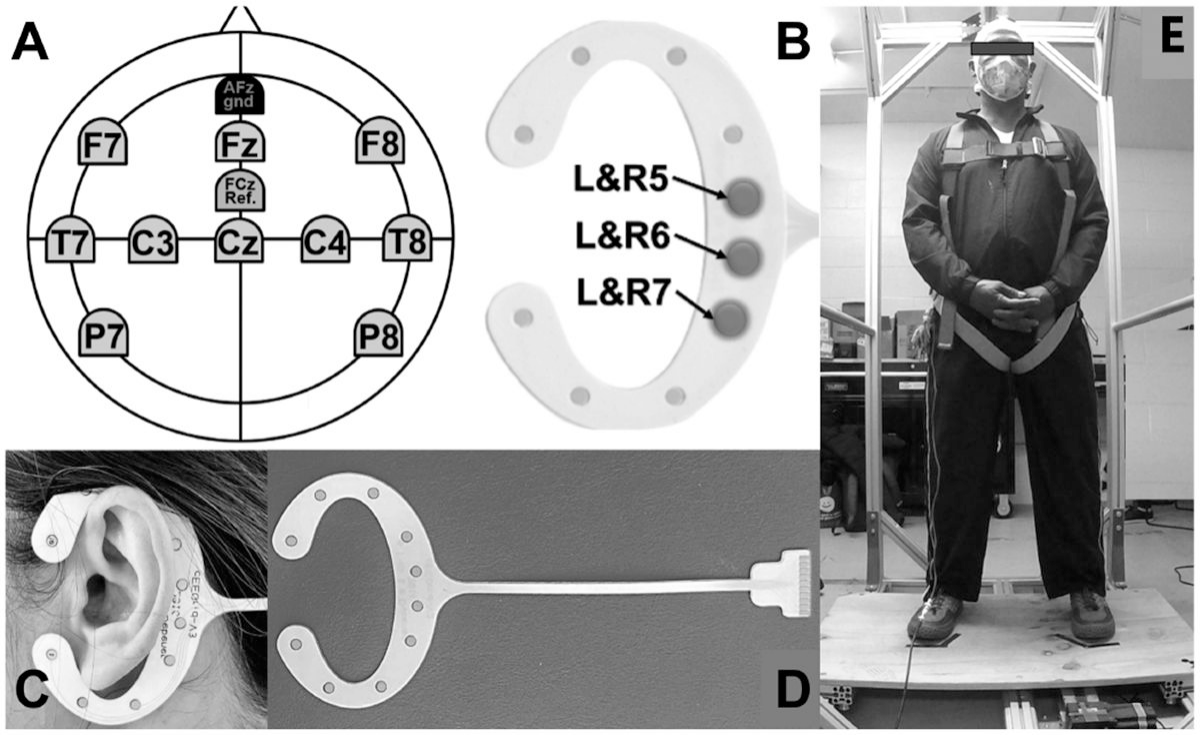
(A) Scalp EEG channels. (B) mastoid channel placements. (C and D) cEEGrids. (E) Illustration of experimental setup.

Participants were instrumented with 10-channel scalp active AgCl electrodes over an EasyCap (Brain Vision, NC), placed at Fz, Cz, C3, C4, F7, F8, T7, T8, P7 and P8 according to the 10-20 system. Additionally, three ear channels (L5-L7 and R5-R7) from cEEGrids (TMSI, Netherlands) were patched over each mastoid, with reference and ground electrodes at FCz and AFz, respectively (Fig. 1a-d). All EEG electrodes were connected to a 16-channel Brain Vision LiveAmp system. Raw EEG data was recorded at 1,000Hz along with 3-axis accelerations, filtered offline with a 2nd order Butterworth bandpass filter (2.5Hz-30Hz) and a 60Hz notch filter. The platform acceleration was recorded at 150Hz (using Delsys Inc.) and synchronized with the EEG data using a Transistor-transistor logic (TTL) pulse.

Data was collected while the subjects stood quietly on a 60×90cm custom-built platform with feet shoulder-width apart and eyes open (Fig. 1e). Rapid translations of the platform of 20cm displacement at 100cm/s and 0.2g acceleration were administered. The platform returned to its starting position after each perturbation with 7s intervals for foot adjustment. The distribution of trials in this study was designed to examine the influence of expectation levels on participants’ postural responses. The trial distribution consisted of 34 trials, with the first 15 trials being posterior translations, followed by an anterior translation as the 16^th^ trial. This was followed by another series of posterior trials (trials 17-21) and interspersed anterior trials (trials 22, 28, and 29). The final set of trials consisted of posterior translations (trials 30-33) and ended with an anterior translation as the 34th trial. This trial sequence aimed to allow participants to become accustomed to the perturbation pattern initially and then introduce unexpected perturbations to capture spontaneous reactions and potential startle responses. Participants were unaware of the total number of trials or the specific order, ensuring unbiased and natural responses.

The utilization of the protocol involving perturbations in various directions has been extensively employed in psychological and motor studies centered around prediction [18]. Although directional parameters will alter the specific muscular activation (synergy) selected to respond to the disturbance [27], they are unlikely to alter the descending cortical activity signaling the need for a particular motor module [28]. In addition, prior studies that focused on Cz and Fz signals did not demonstrate any differences with the directionality of the disturbance [15]. The similarity in velocity and distance between perturbations does not raise concerns, as our primary objective was to introduce a single unpredictable parameter (direction) to impose the desired condition.

To minimize anticipation bias, experimenters engaged in casual conversation with the participants, diverting their attention and promoting a relaxed state. This conversational distraction aimed to ensure that the participants were not anticipating the onset of the first perturbation. During this initial conversation, the first platform translation was initiated unexpectedly, leading to a pronounced startle response from the participants. No additional conversation took place during subsequent trials. This verbal distraction method aimed to elicit a more natural response to perturbation, reducing the influence of anticipatory processes.

In the protocol, we had six translations considered unpredictable: the first posterior translation and five anterior translations presented at specific points among the total thirty-four perturbations. Translations 2-5 were deliberately excluded from further analysis, leaving the remaining 24 posterior translations considered predictable. The rationale behind this choice was to create a more consistent and controlled environment for participants to adapt to the disturbances. Similar protocols were used in previous studies. For example, in a related previous investigation [25], a similar protocol was adopted by excluding the first five perturbations to investigate central habituation rather than sensory adaptation in postural responses. Additionally, Allum and colleagues conducted further studies [29], [30], [31] exploring the changes that occur in the first five trials. This exclusion helped minimize potential confounding effects introduced by the unpredictable translations, enabling us to investigate the predictable components of postural responses more precisely.

### C. Data Analysis

The onset of each of the 30 perturbation events was determined using the platform accelerometer, specifically by identifying the first noticeable burst of activity recorded by the accelerometer. For each event, a 300 ms EEG epoch following the onset was considered as the event EEG epoch. Out of a total of 1,200 event EEG epochs per EEG channel that were planned for the 40 participants in this study, 40 events could not be completed due to technical issues, resulting in a total of 1,160 events analyzed. All event epochs were detrended and baseline-corrected based on the average and standard deviation of EEG magnitude from 300 ms to 100 ms prior to the event onset to remove any linear trend or DC shifts. To facilitate comparison across subjects, within each channel, the EEG epochs were then normalized by treating the maximum EEG magnitude of each subject’s first event epoch as a reference point and scaling the remaining of the subject’s EEG epochs proportionally.

In this study, we employed the Root-Mean-Square (RMS) analysis, a statistical measure commonly used to quantify the magnitude or intensity of a varying signal or data set. The application of RMS analysis in EEG research has a long-standing history, and it has been utilized in numerous studies [32], [33], [34] utilizing this technique to explore brain dynamics and related phenomena based on EEG data.

For our dataset, RMS values of each EEG epoch were calculated for each channel and each subject by taking the square root of the mean of the squared values of the EEG. The average of the RMS values across “unpredictable” and “predictable” events was computed for each channel from each subject. Analysis was centered around the midline cortical electrode sites at Cz and FCz, as a previous study [15] revealed these sites to exhibit the most pronounced cortical response to postural perturbations. Additionally, mastoid channels were also examined to assess the reliability of mastoid EEG signals in responding to postural perturbations. As high lateral correlations were also reported in [35], the average of the three mastoid channels from each ear was used.

RMS responses from the cortical channels and mastoid channels were compared with Repeated-Measure mixed model 3-way ANOVAs to examine the effect of age (young vs older adults), sex (males vs females), and perturbation type (unpredictable and predictable). Pairwise comparisons were performed with Tukey’s HSD test with a p-level set at 0.05. The effect size was calculated with Cohen’s D.

## III. Results

**Fig. 2.**
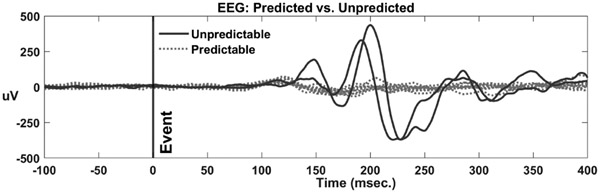
Exemplar EEG activities during two unpredictable events (trials 1 and 16) and ten predictable events (trials 6-15).

**Fig. 3.**
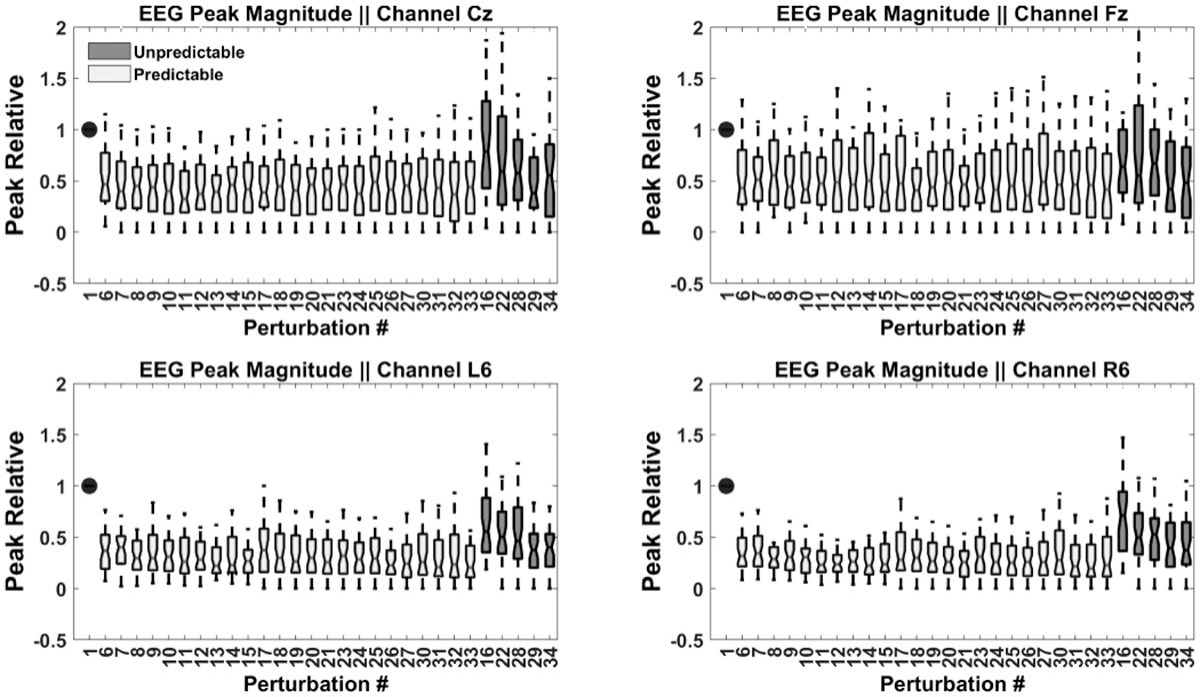
EEG peak magnitudes comparison across cortical and mastoid channels. The boxplot represents predictable events (light-colored) and unpredictable events (dark-colored). The first event is depicted as a single solid dark dot, serving as the reference point for normalization of all events.

EEG RMS responses were examined for each subject across all trials and revealed considerably greater amplitudes during unpredictable compared to predictable disturbances (Fig. 2). Although individual onsets varied, the onset of the larger unpredictable response always appeared within 150 ms of the postural disturbance supporting its description as a PEP. Magnitude differences due to task were evident at each electrode location (Fig. 3). The reported EEG peak magnitudes represent the highest positive peak identified within the analyzed EEG epoch without specifically considering component designations such as P1, N1, P2, or N2, aiming to capture the most prominent positive deflection occurring within a 300-millisecond time window following the onset of the perturbation.

**Fig. 4.**
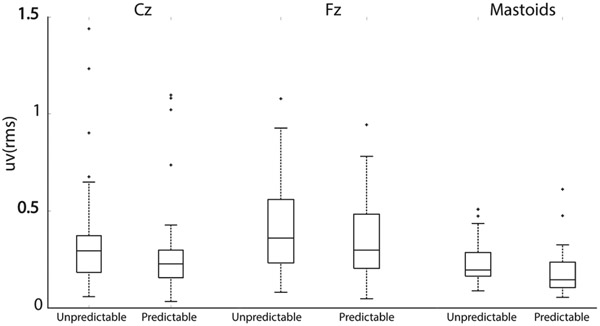
Boxplot of the RMS responses from Cz, Fz, and mastoid EEG sensors (EEG sensors on the mastoid process behind the ears averaged) showing the statistically significant main effect of prediction across all EEG channels. Straight line within the box corresponds to the median, the lower and upper box edges correspond respectively to the 25^th^ and 75^th^ the interquartile ranges (IQR). The whiskers correspond to the minimum and maximum values of the distribution and the crosses are outlier defined as value above 1.5xIQR away from the top or bottom of the box.

**Fig. 5.**
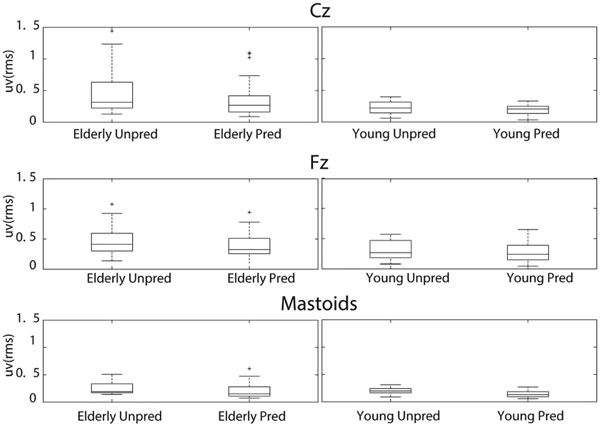
Box plot of the RMS responses from Cz (up) Fz (middle), and mastoid EEG (bottom) sensors (EEG sensors on the mastoid process behind the ears averaged), reported for perturbation type in both age groups.

**Fig. 6.**
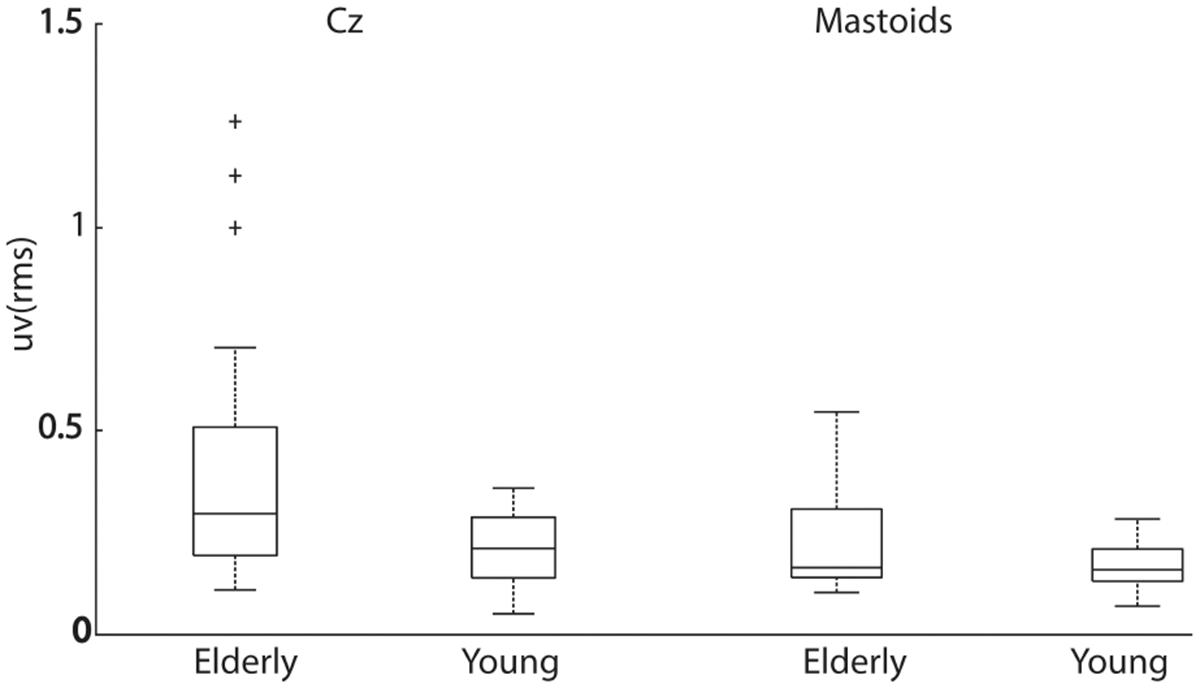
Box plot of the RMS responses from Cz (left), and mastoid EEG (right) sensors (EEG sensors on the mastoid process behind the ears averaged), reported for both age groups.

**Fig. 7.**
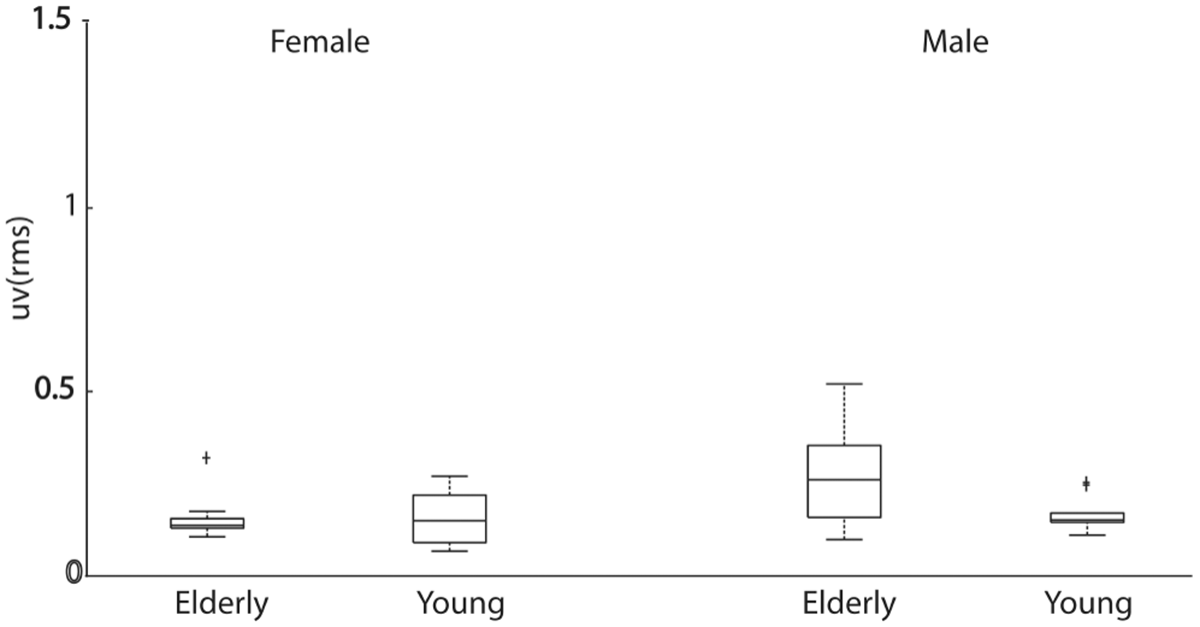
Box plot of the RMS responses from Females (left), and Males (right) participants, reported for both age groups.

**Fig. 8.**
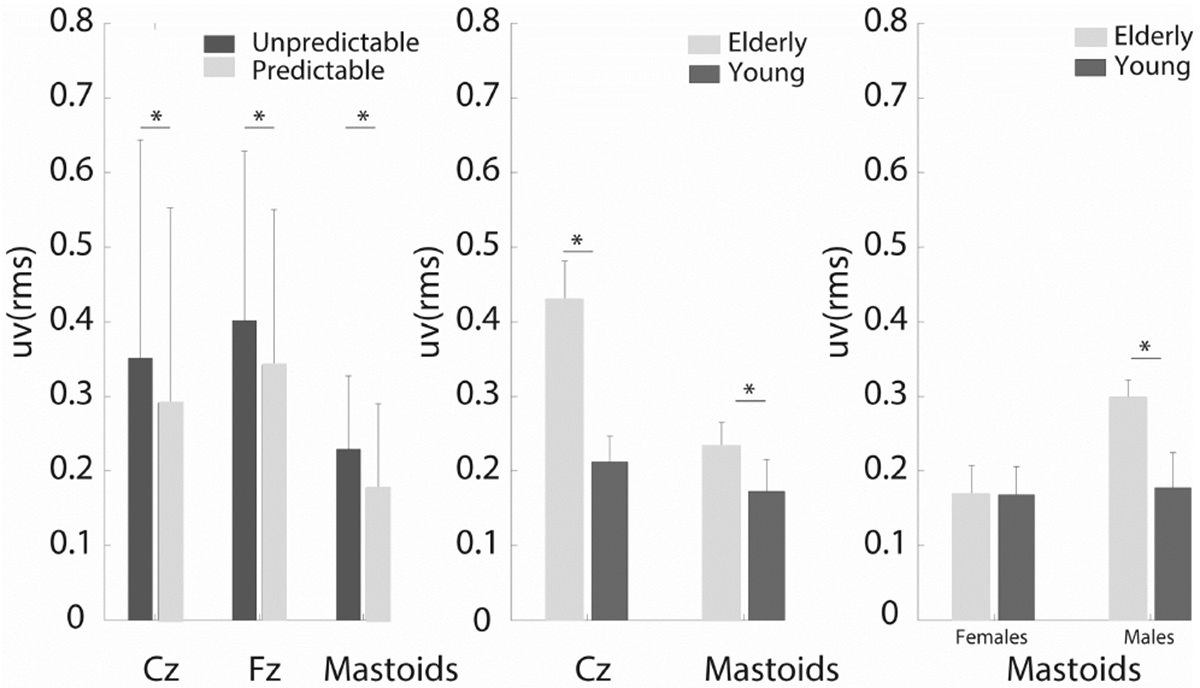
RMS responses from Cz, Fz, and mastoid EEG sensors reveal effects of prediction (left), age (middle), and sex (right).

Statistically significant main effects of perturbation type showed that EEG RMS responses were significantly greater with unpredictable disturbances at each electrode location: Cz (F_(1,34)_ = 12.7, p<0.001, d = 0.2), Fz (F_(1,34)_ = 10.5, p<0.003, d = 0.3) and mastoid (F_(1,36)_ = 68.2 p<0.0001, d = 0.5) (Fig. 4). There was no statistically significant interaction between age group and perturbation type across all channels (Cz: F(1,34)=0.5, p=0.5, Fz: F(1,34)=10.5, p=0.3, Mastoids: F(1,36)=1.8, p=0.2) as the EEG RMS responses were greater in the unpredictable conditions in all age groups across all channels (Fig. 5). EEG RMS responses were also significantly greater in elderly than in young adults at the Cz (F_(1,34)_ = 6.6, p<0.02, d=5.1) and mastoid locations (F_(1,34)_ = 4.6, p<0.04, d=1.6) (Fig. 6). A gender effect also appeared at the mastoid sensor location (Fig. 7) (F_(1,36)_) = 6.1, p<0.04 d=1.9). A statistically significant interaction effect (F_(1,36)_ = 4.47, p=0.04) (Fig. 7) suggests that the responses from elderly male adults were responsible for the observed age effect (Tukey’s HSD, p<0.03 d=3.3). The differences between the means that correspond to the statistical results above are shown in Fig. 8.

## IV. Discussion

Maintaining balance is not a simple, autonomous motor output. Rather, it is a component of each cognitive-motor task that relies on the momentary affective sensory, motor, and cognitive resources [23]. Thus, it is not surprising that balance mechanisms will vary depending on the predictability of a task [36]. Falls occur most frequently when tasks have unanticipated components, such as unexpected obstacles or forces, and the elicitation of the motor behavior is strongly influenced by the performer’s ability to prepare and optimize motor responses to instability. The impact of expectation on response magnitude suggests that unpredictable tasks elicit additional response components. In the context of this scenario, it is plausible to consider that the increased activation observed could be related to the startle response [25]. The startle reflex is a fast, protective reflex response of the muscular system to loud noise or other intensive surprising stimuli. In humans, the addition of a startle response to the automatic postural responses has been shown to have beneficial properties such as accelerated latencies [37] and magnified postural reactions [29], [30], [38]. However, it is important to note that this is only a suggestion based on the observed findings, and further investigation would be necessary to establish conclusive evidence regarding the specific mechanisms underlying the increased activation in response to unpredictable tasks.

The present study investigated whether a single recording site could be used to signify the neural activity elicited by unpredictable postural perturbations that produce a loss of balance. Identifying a recording site that would reliably report neural activity that characterizes a forthcoming loss of balance would be a valuable marker for the development of future technologies focused on preventing falls or injury resulting from falls.

Our protocol was structured so that the direction of the disturbance was unpredictable. While all trials were designed to have the same predictability in timing (i.e., the unpredictable perturbations occurred irregularly within the trial sequence), the direction of the perturbation changed from posterior to anterior in trial 16, creating an unexpected directional demand on the participants. It is, of course, possible that the observed change in neural activation was due to the need for different postural behaviors required in each spatial direction. Directional tuning, however, occurs principally in the pre-motor and primary motor cortex [39] and is characterized by the groupings of cells activated in those areas rather than by the magnitude of global activation.

We found that, as previously described [40], unpredictable perturbations elicited significantly larger magnitudes of EEG activity than predictable perturbations at each sampled electrode location (Cz, Fz, and mastoid). Although the activation affected by the task constraint was observed at all recording sites, recordings at the mastoid sensors were particularly evident and have the additional benefit of being less impacted by additional cortical tasks [41] when only postural behaviors are of interest. Therefore, we would argue that signals obtained at this recording site are more reliable indicators of the neural activity elicited by instability. However, this is hypothetical, and more research is needed to confirm our interpretation of this result.

Sex emerged as a significant biological variable in these data. Elderly male adults exhibited significantly greater magnitudes at the mastoid sensor than both young adults and elderly females. Because elderly females have been reported as being treated more frequently for falling injuries than elderly males [42], these results could be interpreted as a need for greater global activation with aging in order to produce effective stabilizing behaviors. A functional outcome to this difference in subcortical activation might indicate that elderly males have increased sensitivity to labyrinthine acceleration signals in order to continue to produce effective postural behaviors to an unexpected disturbance when muscle activation has reduced with age.

A recent paper [43], however, suggests that pain and comorbidity increase the risk of falls in men more than in women, and the increased neural activation could be reflective of additional confounding factors. Therefore, elderly males may become more reactive to unexpected events than elderly females as a result of increased sedentary behavior with age [44]. Clearly, these hypotheses are purely speculative and need to be further explored if we are to fully understand how the startle reflex participated in fall recovery behaviors.

While our study suggests a positive outcome of incorporating EEG data into fall detection systems to identify the presence of a postural perturbation and predict falls, it is important to acknowledge the inherent limitations. To begin with, the small dataset could potentially limit the applicability of our findings to wider populations. It is important to acknowledge that our study primarily focused on functional and healthy elderly participants. Including individuals with diverse cognitive profiles (e.g., Alzheimer’s disease, Parkinson’s disease), cognitive deficits (e.g., memory loss, trouble concentrating), or other physical conditions (e.g., hip fracture history) would provide a more comprehensive accommodation of the needs of various populations.

Although there is potential for conversational speech to alter attentional and cognitive demands [45], we were careful to cease the conversation as soon as the platform motion was initiated. Our intention was to distract the participant to minimize anticipation of the platform motion in the first trial. We acknowledge, however, that this aspect of our methodology could be viewed as an additional variable in the first trial.

Our investigation also exclusively relied on a specific type of postural perturbation collected in a controlled laboratory environment without the inclusion of real falls. This absence of real-world fall incidents may reduce the ecological validity of our findings and their direct applicability to practical scenarios. Future research endeavors should consider incorporating various types of actual falls and daily activities to provide a more comprehensive understanding of fall detection and enable the development of more effective solutions.

Notwithstanding these limitations, identifying the alteration of EEG responses associated with balance adjustments under different expectation levels will help differentiate between accidental falls and self-initiated activities that resemble falls in daily life. Incorporating such EEG data into fall detection methodologies should facilitate more accurate prediction of falls.

## V. Conclusion

In summary, we have identified EEG signals that indicate a shift from a stable to an unstable state with high accuracy in both healthy young and elderly adults [46]. Our findings suggest that the predictability of a balance disturbance can significantly change brain activity: unpredictable perturbations elicit greater magnitudes of EEG activity than predictable perturbations. This difference in neural activity may be attributed to the startle response, which is known to accelerate movement latencies [37] and exaggerate motor reactions to balance perturbation [29], [30], [38]. Moreover, our study revealed sex differences in the magnitudes of EEG activity, with elderly males exhibiting greater activation at the mastoid sensor than both young adults and elderly females. Sex and age differences in EEG balance-related activity suggest that fall detection devices may need to be tailored to specific populations and that one solution may not fit all. Overall, these results provide valuable insights into the neural mechanisms underlying postural control and may have implications for future technologies aimed at preventing falls or injuries resulting from falls.
